# Zn^2+^-Mediated Co-Deposition of Dopamine/Tannic Acid/ZIF-8 on PVDF Hollow Fiber Membranes for Enhanced Antifouling Performance and Protein Separation

**DOI:** 10.3390/membranes15090277

**Published:** 2025-09-15

**Authors:** Lei Ni, Qiancheng Cui, Zhe Wang, Xueting Zhang, Jun Ma, Wenjuan Zhang, Caihong Liu

**Affiliations:** 1 School of Material Science and Engineering, Tiangong University, Tianjin 300387, China; 2 Tianjin Key Laboratory of Aquatic Science and Technology, School of Environmental and Municipal Engineering, Tianjin Chengjian University, Tianjin 300384, China; 3 State Key Laboratory of Urban Water Resource and Environment, Harbin Institute of Technology, Harbin 150090, China; 4 Key Laboratory of Eco-Environments in Three Gorges Reservoir Region, Ministry of Education, College of Environment and Ecology, Chongqing University, Chongqing 400044, China

**Keywords:** hollow fiber membrane, dopamine, tannin, ZIF-8, antifouling

## Abstract

The inherent hydrophobicity of poly(vinylidene fluoride) (PVDF) ultrafiltration membranes leads to severe membrane fouling when processing proteinaceous solutions and organic contaminants, significantly limiting their practical applications. This study presents a novel metal-ion mediated co-deposition strategy for fabricating high-performance antifouling poly(vinylidene fluoride) (PVDF) hollow fiber ultrafiltration membranes. Through Zn^2+^ coordination-driven self-assembly, a uniform and stable composite coating of dopamine (DA), tannic acid (TA), and ZIF-8 nanoparticles was successfully constructed on the membrane surface under mild conditions. The modified membrane exhibited significantly enhanced hydrophilicity, with a water contact angle of 21° and zeta potential of −29.68 mV, facilitating the formation of a dense hydration layer that effectively prevented protein adhesion. The membrane demonstrated exceptional separation performance, achieving a pure water permeability of 771 L/(m^2^∙h∙bar) and bovine serum albumin (BSA) rejection of 97.7%. Furthermore, it showed outstanding antifouling capability with flux recovery rates exceeding 83.6%, 74.7%, and 71.5% after fouling by BSA, lysozyme, and ovalbumin, respectively. xDLVO analysis revealed substantially increased interfacial free energy and stronger repulsive interactions between the modified surface and protein foulants. The antifouling mechanism was attributed to the synergistic effects of hydration layer formation, optimized pore structure, additional water transport pathways from ZIF-8 incorporation, and electrostatic repulsion from negatively charged surface groups. This work provides valuable insights into the rational design of high-performance antifouling membranes for sustainable water treatment and protein separation applications.

## 1. Introduction

Ultrafiltration (UF) membranes, with pore sizes ranging from 2 to 100 nm, enable the effective removal of contaminants of microorganisms, natural organic matters, colloidal substances, et al., from sewage [1]. Among UF materials, poly(vinylidene fluoride) (PVDF) stands out due to its exceptional mechanical strength and chemical stability and has been extensively investigated and applied in water treatment [2,3,4,5]. However, the inherent hydrophobicity of PVDF membranes induces high interfacial energy at membrane–water interfaces, promoting severe fouling during proteinaceous or organic pollutant filtration. This fouling manifests as pore blockage and surface accumulation, leading to reduced porosity, declined flux, increased energy consumption, and shortened membrane lifespan [6], ultimately limiting PVDF’s applications in the food, pharmaceutical, and wastewater treatment sectors [7,8]. Traditional fouling mitigation strategies (e.g., pre-oxidation, coagulation, cleaning procedures) often introduce harmful by-products and elevate operational costs [9,10]. Alternatively, constructing a hydrophilic surface hydration layer can minimize foulant–membrane interactions during initial fouling stages [11]. Recent approaches focus on modifying membranes with hydrophilic moieties (e.g., –OH, –NH_2_), inorganic nanoparticles, metal-organic frameworks (MOFs), or zwitterionic polymers. MOFs are novel nanoporous particles consisting of metal ions and organic ligands, and exhibiting excellent compatibility with the organic matrix [12,13,14]. Zeolitic imidazolate framework-8 (ZIF-8), a Zn-based MOF with tunable pores and exceptional stability, has shown promise in PVDF membrane modification [15]. Karimi et al. [16] formed a uniform ZIF-8 layer on a PVDF ultrafiltration membrane, which can significantly improve the water flux and flux recovery rates (FRRs) to 134 L/(m^2^ h) and 71.4%, respectively. Guo et al. [17] in situ modified PVDF membrane with a 2 μm ZIF-8 layer obtaining a flux of 137 L/(m^2^ h) and protein rejection of 97.5%. However, the stability of nanoporous particles on the UF membrane surface needs to be further improved through other schemes.

Dopamine (DA) exhibits unique adhesion to low-surface-energy polymers (e.g., PVDF, polypropylene) via self-polymerization into polydopamine (PDA), which enhances wettability and biocompatibility through catechol/amine groups [18,19,20,21,22]. However, the DA self-polymerization process is liable to generate large aggregates through non-covalent effects impacting the membrane surface morphology [23,24,25]. Recent advances have demonstrated accelerated deposition strategies using various catalysts or energy inputs. However, these methods may have their own limitations in terms of coating uniformity and stability. In addition, its residual hydrophobic aromatic domains limit hydrophilicity improvements. Notably, PDA’s phenolic hydroxyl and amino groups enable secondary reactions (e.g., Michael addition/Schiff base reactions) with –NH_2_/–OH/–COOH-containing molecules under an aerobic weakly alkaline condition [26,27,28], offering avenues for further hydrophilicity enhancement [29,30]. For instance, Zin et al. [31] achieved a super-hydrophilic membrane surface with high oil rejection (>98%) by co-depositing DA/polyethyleneimine on a PVDF microfiltration membrane, while Ren et al. [32] combined PDA with 2-*N*-propylsulfonated chitosan (PCS) to reduce the water contact angle to 39° and achieve 90.4% flux recovery. Tannic acid (TA) is a natural reducing agent with five catechol groups in one TA molecule, and can form more viscous networks than PDA. In this case, compared with the PDA deposition alone, involving TA in the modification process could theoretically improve the adhesion of the modified layer [33]. However, TA’s poor self-polymerization necessitates amines (e.g., PEI) or metal ions (e.g., Fe^3+^, Zn^2+^) as catalysts [34,35]. Moreover, incorporating nanomaterials into DA/TA layers can reduce cross-linking density while creating additional water transport pathways, mitigating the permeability-selectivity trade-off [36,37].

The hollow fiber membrane (HFM) exhibits greater self-supporting capability owing to its unique tubular hollow structure, which distinguishes it from conventional flat membranes. A high packing density and specific surface area can be readily achieved through simple stacking of hollow fiber filaments—advantages not attainable with ordinary flat membranes [38,39,40]. However, unlike flat membranes, hollow fibers possess cylindrical and curved surfaces. Consequently, established modification methods designed for flat membranes are not easily transferable to HFMs, often resulting in uneven coating during modification processes, and in some cases, failure to achieve adequate adhesion of the modified layer. Moreover, DA/TA/ZIF-8 composite modifications for PVDF HFMs and their antifouling mechanisms remain unexplored. This study demonstrates a facile co-deposition strategy conducted at room temperature for PVDF HFMs. The Zn^2+^-mediated chelation with DA/TA accelerates metal-polyphenol network formation and the DA/TA interlayer regulates the ZIF-8 crystallization on the membrane surface, synergistically improving the hydrophilicity and antifouling performance. The physico-chemical properties of membranes before and after modification were thoroughly characterized and analyzed through scanning electron microscopy (SEM), X-ray photoelectron spectroscopy (XPS), attenuated total reflection-Fourier transform infrared spectroscopy (ATR-FTIR), water contact angle (WCA) measuring equipment, and zeta potentiometer. ATR-FTIR spectra were normalized before the data analysis. Filtration performance was evaluated using bovine serum albumin (BSA), lysozyme (LYZ), and ovalbumin (OVA), and the obtained DA/TA/ZIF-8-UF membrane demonstrated superior filtration performance, including high water permeability and excellent protein rejection rates. In addition, antifouling tests against BSA, LYZ, and OVA revealed significantly improved fouling resistance and cleaning recovery properties. The extended Derjaguin–Landau–Verwey–Overbeek (xDLVO) theory analysis provided deep insight into the interfacial interaction energies, quantitatively explaining the antifouling mechanism by revealing reduced adhesion energy between the membrane surface and foulants. This work provides an efficient and scalable surface engineering strategy for constructing multifunctional coatings on hollow fiber membranes, overcoming the limitations of conventional modification techniques suited mainly for flat membranes. In addition, it advances the fundamental understanding of hybrid organic–inorganic antifouling layers and offers a practical pathway for developing high-performance HFMs in applications such as wastewater treatment, protein purification, and biomedical separation.

## 2. Materials and Methods

### 2.1. Chemical Reagents and Materials

Commercial hollow fiber PVDF microfiltration membranes (0.2 μm, Tianjin MOTECH, Tianjin, China) were used as substrates for modification. The anti-fouling properties of a commercial hollow fiber UF membrane (PVDF, 30 kDa) from Henan Hongxin Ultrafiltration Membrane Technology Co., Ltd. were measured for comparison. Dopamine hydrochloride (C_3_H_11_NO_2_·HCl, Macklin, Shanghai, China), TA (C_76_H_52_O_46_, Macklin, Shanghai, China), 2-Methylimidazole (Hmim, Aladdin, Tianjin, China), ethanol (Aladdin, Tianjin, China) and zinc nitrate hexahydrate (Zn(NO_3_)_2_·6H_2_O, Aladdin, Tianjin, China) were used as received. Tris buffer with a pH of 8.5 ± 0.1 was prepared from tris (hydroxymethyl)methylaminomethane (THAM, Beijing Solebo Technology, Beijing, China) and dilute hydrochloric acid solution (HCl, 30%, Tianjin, China). BSA, LYZ, and OVA purchased from Aladdin (Tianjin, China) were used for characterization of separation performance and anti-fouling properties of membranes. Deionized (DI) water with a conductivity of 18.2 MΩ cm was produced in the lab.

### 2.2. Membrane Modification

**Figure 1:**
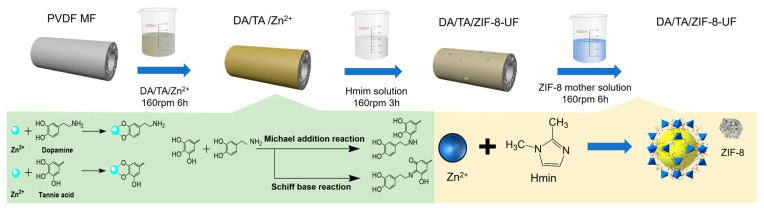
Schematic illustration of membrane modification.

Before modification, the hollow fiber membranes with a length of 10 cm were submerged in the anhydrous ethanol solution for 12 h to remove the residual solvent, and then washed with DI water thoroughly. The moisture on the membrane surface was absorbed by tissue and both ends of each membrane filament were sealed with glue. Then, the membranes were put into the modification solution (DA: 1 g/L, TA: 4 g/L, and Zn(NO_3_)_2_: 0.015 mol/L) and modified for 6 h at room temperature with a shaking speed of 160 rpm. After that, the samples were thoroughly washed with DI water, and activated with 0.7 mmol/L Hmim solution for 3 h following with the ZIF-8 growth process in ZIF-8 mother solution (Zn(NO_3_)_2_·6H_2_O:Hmim:H_2_O =1:75:6000) for 6 h. Finally, the obtained membranes were washed thoroughly using DI water and dried at 60 °C for 12 h. The schematic illustration for the membrane modification process is shown in Figure 1. Table 1 lists the components of modification solutions. The membrane samples were modified in triplicate.

### 2.3. Characterization of Membrane Samples

A field emission SEM (Gemini 500, Zeiss, Germany) was applied for observing the morphology of the membrane surface and cross-sections at a working voltage of 10 kV. All film samples were coated with a thin layer (~10 nm) of gold by sputtering at the current of 15 mA for 60 s prior to field emission SEM testing. Membrane surface functional groups before and after modification were analyzed by an ATR-FTIR spectrometer (Nicolet 6700, Thermo Fisher, Waltham, MA, USA) within the spectral range of 650–4000 cm^−1^. The changes in the membrane surface chemical compositions were determined by an XPS spectrometer (ESCALAB 250Xi, Thermo Scientific, East Grinstead, UK) with a scanning resolution of 0.1 eV. An automatic contact angle meter (DSA100, Kruss, Germany) was used to test the WCA of membrane samples for wettability analysis [41,42]. The hydrophilicity of the whole membrane can be characterized by the water absorption test which was conducted by immersing membranes in DI water for 24 h to obtain wet weight (*M*_wet_) after removing surface water by tissues, and to obtain dry weight (*M*_dry_) after dried at 50 °C for 3 days in an oven. The water absorption rate (WAR) can be calculated from the formula as follows:

(1) $$\mathrm{WAR}=\frac{M_{\mathrm{wet}}-M_{\mathrm{dry}}}{M_{\mathrm{dry}}}\times 100\%$$

The membrane porosity refers to the ratio of the pores’ volume to the total volume of the membrane, which was determined by the weight difference method through immersing of membrane samples in *n*-butanol for 24 h. According to the weight difference in the membrane before and after soaking in *n*-butanol, the membrane porosity (*ε*, %) can be calculated from the following equation:

(2) $$\varepsilon =\frac{\Delta m}{V\cdot \rho }\times 100\%=\frac{m_{1}-m_{0}}{(\pi /4)\left(D^{2}-d^{2}\right)l\rho }\times 100\%$$

where *m*_0_ and *m*_1_ (g) represent the weight of the membrane before and after soaking in *n*-butanol, respectively. *D* (m) and *d* (m) are the outer and inner diameters of the membrane, respectively. *l* (m) is the effective length of the samples, and *ρ* is the density of the *n*-butanol (0.81 g/cm^3^).

### 2.4. Measurement of Membrane Permeability and Protein Rejection

In this study, a self-made cross-flow filtration device with a length of 30 cm and a radius of 1 cm was used to measure the pure water permeability of the membranes. The PVDF HFMs modules were constructed by sealing 6 hollow fibers (an effective area of 20 cm^2^) in a polyurethane (PU) tube and one end of the fibers was sealed with glue. The pressurized DI water permeated from the outside to the inside of the hollow fiber membrane and discharged from the PU tube. The membrane samples were pre-compacted at 4 bar for at least 2 h to stabilize the membrane performance, and then the membrane permeability was measured at 1 bar with the crossflow velocity fixed at 15.0 cm/s. The water permeability (*P*, L/(m^2^∙h∙bar)) can be determined using the following formula:

(3) $$P=\frac{V}{\mathrm{AtP}}$$

where *V* (L) represents the filtration volume, and *A* (m^2^), *t* (h), and *P* (bar) are the effective membrane area, filtration time, and the filtration pressure, respectively.

BSA, LYS, and OVA aqueous solutions with a concentration of 1 g/L were prepared for estimation of the membrane separation performance. The protein rejection (*R*, %) can be calculated via the formula as follows:

(4) $$R=\left(1-\frac{C_{p}}{C_{f}}\right)\times 100\%$$

where *C*_p_ and *C*_f_ (g/L) are the protein concentrations of permeate and feed solutions, respectively. The protein concentration was measured through a UV–Vis spectrophotometer at different wavelengths as shown in Table S1.

The membrane stability was tested through water flushing experiments [43] by stirring membrane samples in DI water on a magnetic stirring apparatus at a rotation speed of 1300 rpm for 24 h. And then the water permeability and WCA at different times were tested.

### 2.5. Organic Fouling Tests

BSA, LYS, and OVA are three different types of proteins applied to investigate the anti-fouling properties of membrane samples, and the molecule properties are listed in Table S1 in supplementary information (SI). Before tests, the membranes were firstly compacted in DI water with the cross-flow filtration device at 4 bar for 2 h, and then the membrane organic fouling tests were carried out through the following procedure two times: (1) filtration with deionized water for 1 h to determine the pure water flux (F_W1_), (2) filtration of the 1 g·L^−1^ protein solution for 2 h to determine the protein fouling permeate flux (F_BSA_, F_LYZ_, and F_OVA_), (3) backwashing with pure water for 1 h under 1 bar, and (4) filtration with deionized water for 1 h after backwashing to determine the pure water flux (F_W2_). The parameters of flux recovery rate (FRR), total fouling rate (R_t_), reversible fouling rate (R_r_), and irreversible fouling rate (R_ir_) were calculated as follows [22]:

(5) $$\mathrm{FRR}=\frac{F_{w2}}{F_{w1}}\times 100\%$$

(6) $$R_{t}=\frac{F_{w1}-F_{x}}{F_{w1}}\times 100\%$$

(7) $$R_{r}=\frac{F_{w2}-F_{x}}{F_{w1}}\times 100\%$$

(8) $$R_{\mathrm{ir}}=\frac{F_{w_{1}}-F_{w2}}{F_{w1}}\times 100\%$$

where *F*_x_ refers to the permeate flux of the contaminants (F_BSA_, F_LYZ_, and F_OVA_).

### 2.6. Analysis of Membrane Surface Anti-Protein Fouling Mechanism

To analyze the anti-protein fouling mechanism of the modified membrane, the adhesive free energies of the membrane-contaminant and the contaminant-contaminant were calculated using the xDLVO theory. [44] From the xDLVO theory, the total interaction energy (Δ$G_{\mathrm{mlf}}^{\mathrm{TOT}}$) between pollutants and the membrane is composed of van der Waals interaction energy (LW, Δ$G_{\mathrm{mlf}}^{\mathrm{LW}}$), electrostatic interaction energy (EL, Δ$G_{\mathrm{mlf}}^{\mathrm{EL}}$), and polar interaction energy (Δ$G_{\mathrm{mlf}}^{\mathrm{AB}}$) [45,46] as expressed in the following formulas:

(9) $$\Delta G_{\mathrm{mlf}}^{\mathrm{TOT}}=\Delta G_{\mathrm{mlf}}^{\mathrm{LW}}+\Delta G_{\mathrm{mlf}}^{\mathrm{EL}}+\Delta G_{\mathrm{mlf}}^{\mathrm{AB}}$$

(10) $$\Delta G_{\mathrm{mlf}}^{\mathrm{LW}}=2\left(\sqrt{\gamma_{l}^{\mathrm{LW}}}-\sqrt{\gamma_{m}^{\mathrm{LW}}}\right)\left(\sqrt{\gamma_{f}^{\mathrm{LW}}}-\sqrt{\gamma_{l}^{\mathrm{LW}}}\right)$$

(11) $$\Delta G_{mlf}^{AB}=2\sqrt{\gamma_{l}^{+}}\left(\sqrt{\gamma_{m}^{-}}+\sqrt{\gamma_{f}^{-}}-\sqrt{\gamma_{l}^{-}}\right)+2\sqrt{\gamma_{l}^{-}}\left(\sqrt{\gamma_{m}^{+}}+\sqrt{\gamma_{f}^{+}}-\sqrt{\gamma_{l}^{+}}\right)-2\left(\sqrt{\gamma_{m}^{+}\gamma_{f}^{-}}+\sqrt{\gamma_{m}^{-}\gamma_{f}^{+}}\right)$$

(12) $$\Delta G_{\mathrm{mlf}}^{\mathrm{EL}}=\frac{\varepsilon_{0}\varepsilon_{r}\kappa }{2}\left(\zeta_{m}^{2}+\zeta_{f}^{2}\right)\left[1-\cot h\left(\kappa d_{0}\right)+\frac{2\zeta_{m}\zeta_{f}}{\zeta_{m}^{2}+\zeta_{f}^{2}}\csc h\left(\kappa d_{0}\right)\right]$$

where the subscripts “*f*, *l*, *m*” denote foulant, water, and membrane, respectively. *ε*_0_ represents the vacuum permittivity, *ε*_r_ represents the relative dielectric constant of aqueous solution, *κ* represents the reciprocal of the Debye constant, and *ζ*_m_ and *ζ*_f_ are zeta potentials of membrane surface and pollutant, respectively [47]. The surface tension parameters of $\gamma^{\mathrm{LW}}$, $\gamma^{-}$, and $\gamma^{+}$ correspond to the tension component of Vander surface, electron donor, and electron acceptor, respectively, and can be obtained from the following equations:

(13) $$\left(1+\cos\theta \right)\gamma_{l}^{\mathrm{TOT}}=2\left(\sqrt{\gamma_{s}^{\mathrm{LW}}\gamma_{l}^{\mathrm{LW}}}+\sqrt{\gamma_{s}^{+}\gamma_{l}^{-}}+\sqrt{\gamma_{s}^{-}\gamma_{l}^{+}}\right)$$

(14) $$\left\{\begin{array}{c} \left(1+\cos\theta_{W}\right)\gamma_{W}^{\mathrm{TOT}}=2\left(\sqrt{\gamma_{s}^{\mathrm{LW}}\gamma_{W}^{\mathrm{LW}}}+\sqrt{\gamma_{s}^{+}\gamma_{W}^{-}}+\sqrt{\gamma_{s}^{-}\gamma_{W}^{+}}\right)\\ \left(1+\cos\theta_{G}\right)\gamma_{G}^{\mathrm{TOT}}=2\left(\sqrt{\gamma_{s}^{\mathrm{LW}}\gamma_{G}^{\mathrm{LW}}}+\sqrt{\gamma_{s}^{+}\gamma_{G}^{-}}+\sqrt{\gamma_{s}^{-}\gamma_{G}^{+}}\right)\\ \left(1+\cos\theta_{D}\right)\gamma_{D}^{\mathrm{TOT}}=2\left(\sqrt{\gamma_{s}^{\mathrm{LW}}\gamma_{D}^{\mathrm{LW}}}+\sqrt{\gamma_{s}^{+}\gamma_{D}^{-}}+\sqrt{\gamma_{s}^{-}\gamma_{D}^{+}}\right) \end{array}\right.$$

where the subscripts *s* and *l* represent solid and liquid surfaces, respectively, while *θ*_W_, *θ*_G_, and *θ*_D_ represent the contact angles of water, glycerol, and diiodomethane, respectively.

## 3. Results and Discussion

### 3.1. Membrane Characterization

#### 3.1.1. Morphologies of the Membranes

**Figure 2:**
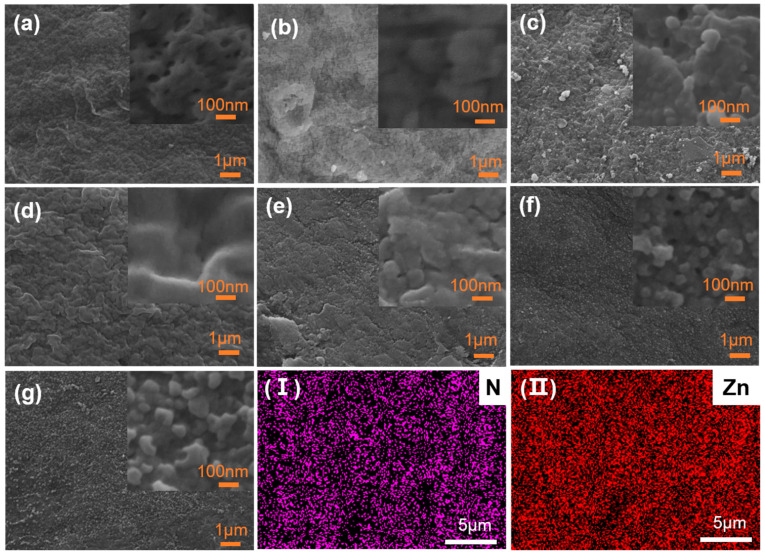
Surface images (**a**–**g**) obtained by SEM for the membranes of (**a**) PVDF substrate, (**b**) TA-UF, (**c**) DA-UF, (**d**) DA/TA-UF, (**e**) TA-ZIF-8-UF, (**f**) DA/ZIF-8-UF, and (**g**) DA/TA/ZIF-8-UF. (**I**) N element and (**II**) Zn element for DA/TA/ZIF-8-UF membrane.

The visual appearance and SEM images of the pristine and modified membranes are shown in Figure S1 and Figure 2a–g. The unmodified PVDF membrane exhibited a white (Figure S1a), porous structure (Figure 2a), whereas the DA-modified membrane (DA-UF) turned dark brown due to the formation of a PDA layer resembling eumelanin (Figure S1a). In contrast, the TA-modified membrane (TA-UF) displayed a light yellow hue characteristic of TA (Figure S1a). When co-deposited, the DA/TA-UF membrane showed an intermediate light yellow tone, suggesting synergistic interactions between DA and TA [48]. Incorporation of ZIF-8 further altered the membrane coloration: the DA/ZIF-8-UF membrane appeared light gray, while the TA/ZIF-8-UF membrane retained a light yellow tint, both reflecting the inherent white color of ZIF-8 nanoparticles (Figure S1a). Notably, the DA/TA/ZIF-8-UF membrane closely resembled the pristine PVDF in color, indicating uniform coating and minimal light scattering (Figure S1a). Cross-sectional SEM images (Figure S1b–h) revealed that all modified membranes retained the original microstructure of the PVDF substrate. The modifiers (DA, TA, and/or ZIF-8) were predominantly deposited on the outer surface, as the sealed ends of the hollow fibers during modification prevented internal penetration. This surface-localized deposition preserved the structural integrity and porosity of the support layer.

Significant morphological differences were observed on the outer surfaces of the modified membranes (Figure 2a–g). The morphology of the TA-UF membrane (Figure 2b) did not change a lot compared to the pristine PVDF (Figure 2a), owing to TA’s limited self-polymerization under alkaline conditions. In contrast, the DA-UF membrane (Figure 2c) displayed unevenly distributed aggregates of varying sizes, characteristic of PDA’s spontaneous self-polymerization [23,49], while the DA/TA-UF membrane (Figure 2d) developed a denser, more continuous, and smoother surface layer, resulting from Michael addition and Schiff base reactions between DA and TA. These reactions promoted a highly cross-linked network with improved adhesion [50]. Incorporation of ZIF-8 further altered surface morphology. The TA/ZIF-8-UF membrane (Figure 2e) showed increased deposition compared to TA-UF, due to Zn^2+^-TA coordination forming a metal-polyphenol network that enhanced TA adhesion. Notably, the DA/TA/ZIF-8-UF membrane (Figure 2g) exhibited a more uniform and continuous ZIF-8 layer with finer crystallites than DA/ZIF-8-UF (Figure 2f), demonstrating that ternary co-deposition yields a homogeneous yet morphologically distinct modification layer without compromising structural integrity. Moreover, the high-resolution SEM image (Figure S2) of the ZIF-8 crystallites on the surface of the DA/TA/ZIF-8-UF membrane reveal the uniformity of deposition. EDX mapping of the DA/TA/ZIF-8-UF membrane (Figure 2I,II) confirmed the uniform distribution of nitrogen (N) and zinc (Zn), characteristic of ZIF-8, further verifying the successful and homogeneous deposition of the composite layer.

#### 3.1.2. Surface Chemical Compositions

**Figure 3:**
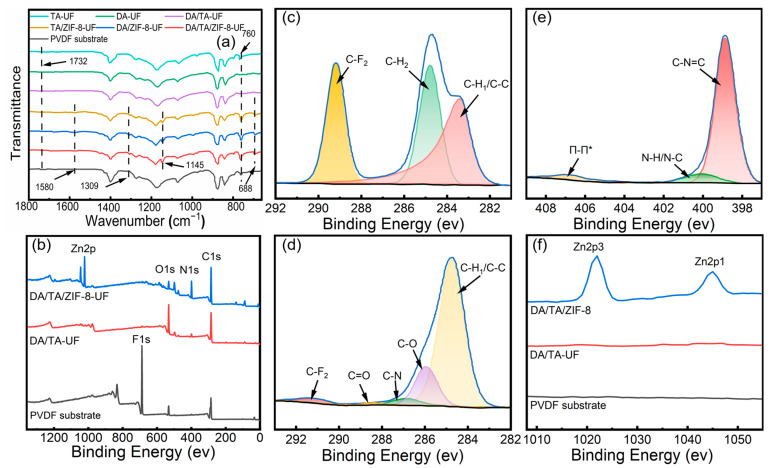
(**a**) ATR-FTIR spectra and (**b**) full-scan XPS spectra of membranes. (**c**,**d**) C1s PVDF substrate and DA/TA/ZIF-8-UF membrane, respectively. (**e**) N1s of DA/TA/ZIF-8-UF membrane. (**f**) Zn2p of membranes before and after modification.

The ATR-FTIR spectra of pristine and modified membranes are presented in Figure 3a and Figure S3. The TA-UF membrane exhibited stronger peak intensities than DA-UF, consistent with TA’s higher catechol content and superior adhesion [33]. The DA/TA-UF membrane (Figure 3a) showed enhanced absorption at 760 cm^−1^ (phenolic O-H out-of-plane bending), attributed to synergistic reactions between TA-derived quinones and DA’s amine groups via Michael addition and Schiff base reactions, alongside DA self-cross-linking, resulting in a highly cross-linked network. With the introduction of ZIF-8, characteristic absorption peaks emerged at 3133 cm^−1^ (C-H stretching vibrations of methyl), 2925 cm^−1^ (C-H stretching vibrations of imidazole rings), 1580 cm^−1^ (the deformation vibration of N-H), 1309 cm^−1^ (asymmetric stretching vibration of C-O-C), 1145 cm^−1^ (C-N stretching vibration in the imidazole ring), and 688 cm^−1^ (out-of-plane bending of the imidazole ring) [51,52]. The DA/TA/ZIF-8-UF membrane exhibited higher intensities for these peaks than TA/ZIF-8-UF or DA/ZIF-8-UF, indicating that the DA/TA network facilitated Zn^2+^ coordination and imidazole ligand binding for formation of metal-phenolic compounds, promoting ZIF-8 nucleation and crystallization. The reduced C=O peak intensity at 1732 cm^−1^ further confirmed ZIF-8 formation on the cross-linked layer [53,54]. Notably, the deposition of ZIF-8 enhances the out-of-plane bending peak of the O-H for the TA phenol hydroxyl group at 760 cm^−1^, evidence of Zn^2+^-TA coordination increasing TA’s cross-linking density [55,56]. Thus, the ternary DA/TA/ZIF-8 system synergistically formed a composite coating layer on the PVDF substrate.

Figure 3b–e and Figure S4 presented XPS spectra of the wide scan and high resolution of C1s, N1s, and O1s of the PVDF substrate, DA/TA-UF, and DA/TA/ZIF-8-UF, with corresponding atomic percentages summarized in Table 2. Compared to the PVDF substrate, the DA/TA-UF membrane exhibited a sharp decrease in fluorine (F) content (from 33.94% to 0.66%), a substantial increase in oxygen (O) content (from 6.87% to 27.44%) and nitrogen (N) content (from 0.97% to 3.93%), confirming the successful surface coating with polar functional groups (–OH, –NH_2_) that enhance hydrophilicity. For the DA/TA/ZIF-8-UF membrane, the F content further decreased to 0.55%, and zinc (Zn) emerged with an atomic percentage of 9.47%, directly evidencing ZIF-8 deposition facilitated by Zn^2+^ coordination with gallate groups in TA. The decrease in the O atomic percentage for the DA/TA/ZIF-8-UF membrane, compared to the DA/TA-UF membrane, is attributed to the surface coverage by the ZIF-8 layer, which has a lower oxygen content relative to the oxygen-rich TA. From the O1s peak fitting (Figure S4), the appearance of a Zn-O peak at 531.2 eV in the DA/TA/ZIF-8-UF membrane provides additional evidence for the formation of the ZIF-8 layer on the membrane surface [57,58]. The high N content (17.15%) originated from imidazole ligands in ZIF-8, further confirming crystalline ZIF-8 formation. High-resolution C1s spectra (Figure 3c,d) revealed a reduced C-F_2_ peak (289.3 eV) in DA/TA/ZIF-8-UF compared to PVDF, alongside new peaks at 286.0 eV (C-O) and 287 eV (C=N), indicative of Schiff base reactions between DA and TA. The C=O peak at 288.2 eV corresponded to quinone/ester groups from DA oxidation [59,60,61], aligning with FTIR-proposed cross-linking mechanisms. In the N1s spectrum (Figure 3e), peaks at 398.8 eV (C–N=C) and 406.8 eV (π-π* bonding) characterized imidazole in ZIF-8. Combined with Zn 2p peaks at 1022 eV and 1046 eV (Figure 3f), these results provide conclusive evidence of ZIF-8 incorporation [62].

#### 3.1.3. Membrane Hydrophilicity and Porosity

**Figure 4:**
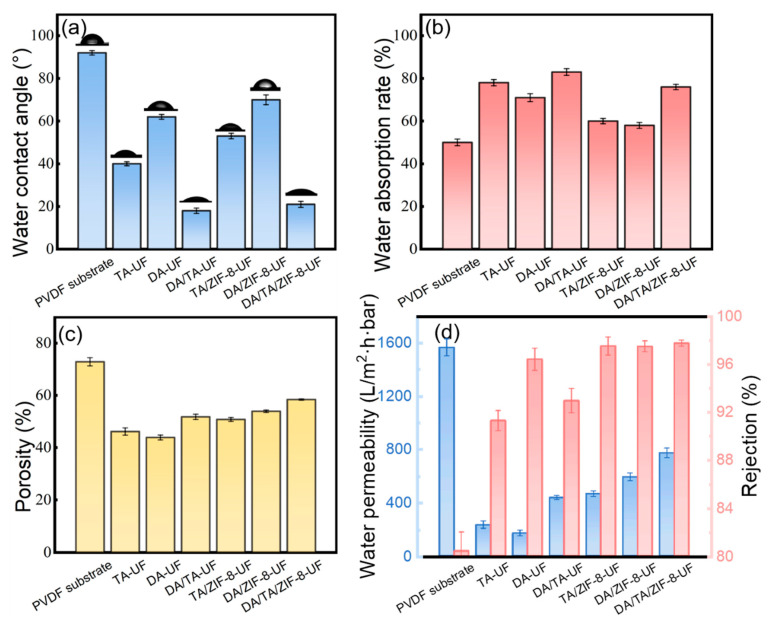
(**a**) Water contact angle, (**b**) water absorption rate, (**c**) porosity, and (**d**) separation performance of membranes before and after modification. Error bars are the standard deviation corresponding to the arithmetic square root of the arithmetic mean of the squared deviations from the mean.

The hydrophilicity of the membranes was evaluated by WCA (surface property) and WAR (bulk property), as shown in Figure 4a,b. The pristine PVDF membrane exhibited strong hydrophobicity (WCA~92°). Surface modification with DA or TA significantly improved hydrophilicity, reducing WCAs to 62 ± 1° and 40 ± 1°, respectively, due to their abundant hydrophilic functional groups. The DA/TA-UF membrane showed a further reduction in WCA (18 ± 1°), demonstrating the synergistic effect of cross-linked networks formed between DA and TA. After incorporating ZIF-8, which exhibits inherent hydrophobicity [63], the WCAs of DA/ZIF-8-UF, TA/ZIF-8-UF, and DA/TA/ZIF-8-UF increased to 70 ± 2°, 53 ± 1°, and 21 ± 1°, respectively. Despite this partial hydrophobization, all modified membranes maintained higher hydrophilicity than pristine PVDF. The WAR results correlated well with surface hydrophilicity trends. The DA-UF, TA-UF, and DA/TA-UF membranes showed WAR values of 78%, 71%, and 83%, respectively, significantly higher than PVDF (50%). With ZIF-8 incorporation, WAR values decreased to 58% (DA/ZIF-8-UF), 60% (TA/ZIF-8-UF), and 76% (DA/TA/ZIF-8-UF), yet remained above the PVDF baseline. The enhanced hydrophilicity of modified membranes reduces interfacial energy with aqueous pollutants, thereby improving antifouling performance.

Membrane porosity, a critical factor governing separation and antifouling performance, was quantitatively evaluated (Figure 4c). The pristine PVDF substrate exhibited a porosity of 72.9%. Modification reduced porosity due to polymer deposition on surfaces and within pores. The DA-UF membrane showed the most significant decline (43.9%), attributed to DA’s strong self-aggregation tendency, which formed dense, pore-blocking networks. In contrast, the DA/TA-UF membrane maintained higher porosity (51.8%), as the Michael addition/Schiff base reactions between DA and TA yielded a more uniform and open cross-linked structure, mitigating pore blockage [48]. Incorporating ZIF-8 increased porosity relative to polymer-only modifications, leveraging its intrinsic nanoporosity. The DA/TA/ZIF-8-UF membrane achieved the highest porosity (58.4%) among modified membranes, exceeding both DA/ZIF-8-UF (50.8%) and TA/ZIF-8-UF (53.8%). This enhancement stems from dual mechanisms: Zn^2+^-TA coordination creating metal-phenolic frameworks, and ZIF-8 crystallization introducing additional void spaces. The synergistic DA/TA system thus facilitates a more porous and functionalized surface architecture.

### 3.2. Separation Performance and Stability of Membranes

**Figure 5:**
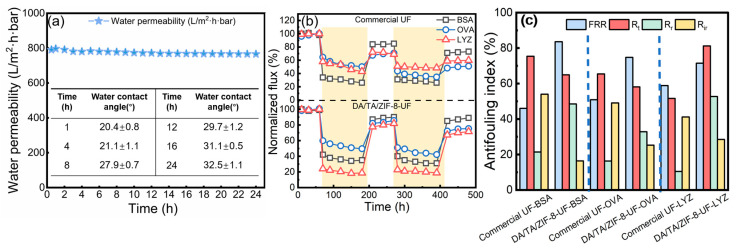
(**a**) Changes of water permeability and water contact angles for DA/TA/ZIF-8-UF membrane in the long-term run. (**b**) The normalized flux variation and (**c**) fouling parameters for DA/TA/ZIF-8-UF membrane and the commercial ultrafiltration membrane.

The water permeability and BSA rejection of the modified membranes are shown in Figure 5c. The pristine PVDF membrane exhibited high water permeability (1565 ± 63 L/(m^2^∙h∙bar)), attributable to its macroporous surface structure (Figure 2a) and high porosity (72.9%, Figure 4c). Surface modification with DA or TA significantly reduced permeability to 171 ± 21 and 233 ± 27 L/(m^2^∙h∙bar), respectively, due to the formation of dense polymer layers (Figure 2e,f) that partially obstructed surface pores. The TA-UF membrane demonstrated higher permeability but lower BSA rejection (91.3 ± 0.8%) compared to DA-UF (96.4 ± 0.9%), consistent with TA’s limited self-polymerization under alkaline conditions, which resulted in incomplete surface coverage and allowed greater water flux through residual macropores. In contrast, the DA/TA-UF membrane showed enhanced permeability (437 ± 16 L/(m^2^∙h∙bar)) relative to DA-UF, owing to improved hydrophilicity (Figure 4a,b) and a more open cross-linked network formed through Michael addition and Schiff base reactions [48,50]. Incorporation of ZIF-8 further improved the separation performance. The DA/TA/ZIF-8-UF membrane achieved a permeability of 771 ± 36 L/(m^2^∙h∙bar) and 97.7 ± 0.2% BSA rejection, representing increases of 76.4% and 5.2% over DA/TA-UF, respectively. This enhancement is attributed to ZIF-8’s nanoporosity, which provides additional water transport pathways while maintaining molecular selectivity, thereby mitigating the permeability–rejection trade-off [3,37,64]. The superior performance of DA/TA/ZIF-8-UF over both DA/ZIF-8-UF and TA/ZIF-8-UF membranes aligns with its higher porosity (Figure 4c) and more uniform surface morphology (Figure 2g). The correlation of membrane permeability–porosity is illustrated in Figure S5, which provides strong support for the performance-structure effects induced by the coating of DA, TA, and ZIF-8. Moreover, the higher rejection rate of BSA than OVA (82.5 ± 0.3%) by DA/TA/ZIF-8-UF membrane in Table S1 was ascribed to the size screening effect, while the lowest rejection rate in LYS (71.4 ± 0.5%) was attributed to the electrostatic attraction effect and size screening effect.

Membrane stability was evaluated through a 24 h hydraulic flushing test (Figure 5a). The DA/TA/ZIF-8-UF membrane maintained stable water permeability throughout, while the water contact angle increased only slightly from 20.4 ± 0.8° to 32.5 ± 1.1°, demonstrating strong physical stability attributed to the robust adhesion of the modification layer through synergistic metal–polyphenol coordination and covalent cross-linking [65]. These results indicate excellent potential for long-term operational stability in protein separation applications.

### 3.3. Antifouling Properties of UF Membranes

The antifouling performance of the DA/TA/ZIF-8-UF membrane was evaluated against BSA, OVA, and LYS (1.0 g/L each) and compared to a commercial PVDF UF membrane (Figure 5b,c). All membranes exhibited significant normalized flux decline due to protein adsorption, with the extent of fouling influenced by protein charge and size. The small decline in flux during each cycle of protein filtration (Figure 5b) demonstrated a formation of a fouling layer on the membrane surface hindering the passage of water molecules. The DA/TA/ZIF-8-UF membrane surface, rich in phenolic hydroxyl groups (pKa ~10), carries a strong negative charge [23]. Consequently, the positively charged LYS experienced severe fouling (R_t_ = 81.2%) due to electrostatic attraction, leading to substantial flux reduction. In contrast, the negatively charged BSA and OVA (Table S1) showed lower total flux decline (R_t_ = 64.9% and 58.1%, respectively) due to electrostatic repulsion. However, the smaller molecular size of OVA enabled deeper pore penetration and formation of a denser, more irreversible fouling layer (R_ir_ = 25.3%) compared to BSA (R_ir_ = 16.4%), as hydraulic cleaning was less effective for internally deposited OVA [66]. Compared to the commercial PVDF membrane, the DA/TA/ZIF-8-UF membrane demonstrated significantly improved antifouling performance across all tested proteins, exhibiting higher flux recovery ratios (FRRs) and reversible fouling ratios (R_rs_), along with lower total (R_t_) and irreversible (R_ir_) fouling ratios. This enhancement is attributed to the hydrophilic coating layer, which forms a robust hydration barrier that effectively shields the surface from protein adhesion. The hydration layer minimizes electrostatic interactions and facilitates foulant removal during cleaning, thereby improving fouling reversibility and maintaining long-term filtration performance.

### 3.4. Anti-Fouling Mechanisms from xDLVO Theory

Membrane fouling is predominantly governed by interfacial interaction energies between foulants and membrane surfaces, which can be quantitatively evaluated using the xDLVO theory [34]. To determine the surface tension parameters, contact angle measurements were performed using three probe liquids: water and glycerol (polar solvents) and dichloromethane (non-polar solvent). The resulting data and zeta potential values are summarized in Table S2 and Table S3. The surface tension components of both the DA/TA/ZIF-8-UF membrane and commercial PVDF UF membrane, along with those of the three protein foulants (BSA, OVA, LYS), are presented in Table 3. The DA/TA/ZIF-8-UF membrane exhibited a significantly higher electron donor parameter (γ^−^) compared to the commercial membrane, consistent with its more negative surface charge (−29.68 mV vs. −19.83 mV). This enhanced negativity originates from the abundant phenolic hydroxyl groups of TA and the quinone groups from oxidized DA, which are deprotonated under near-neutral conditions.

Furthermore, the modified membrane showed higher Lifshitz-van der Waals (γ^LW^) and acid-base (γ^AB^) components than the commercial PVDF UF, due to the introduction of hydrophilic functional groups (e.g., amino, catechol, and hydroxyl groups) from DA and TA. These groups facilitate strong hydrogen bonding with water molecules, promoting the formation of a dense hydration layer on the membrane surface [6,67]. This hydration layer acts as a physical and energetic barrier, reducing the adhesion strength of foulants and enhancing fouling reversibility, as schematically illustrated in Figure 5b,c.

The interfacial free energies between the membrane surfaces and protein foulants were calculated based on xDLVO theory, with results summarized in Table 4. The cohesive free energy (ΔG_SlS_) of LYZ is negative, indicating a strong propensity for self-aggregation and fouling layer formation, while the positive ΔG_SlS_ values for BSA and OVA suggest weaker cohesive tendencies. The total interfacial free energy ($\Delta G_{\mathrm{mlf}}^{\mathrm{TOT}}$) between the foulants and the commercial PVDF membrane is significantly lower than that for the DA/TA/ZIF-8-UF membrane, indicating stronger foulant-membrane adhesion and more severe fouling on the commercial membrane. In contrast, the DA/TA/ZIF-8-UF membrane exhibits substantially higher $\Delta G_{\mathrm{mlf}}^{\mathrm{TOT}}$ values (less attractive/more repulsive) for all three proteins, demonstrating superior antifouling potential. According to the xDLVO theory, a positive value of the total interfacial free energy ($\Delta G_{\mathrm{mlf}}^{\mathrm{TOT}}$) indicates a net repulsive interaction, while a negative value signifies a net attractive interaction, which promotes fouling. Notably, the DA/TA/ZIF-8-UF membrane shows higher polar interaction energy ($\Delta G_{\mathrm{mlf}}^{\mathrm{AB}}$) due to abundant hydrophilic groups, a lower Lifshitz-van der Waals component $\Delta G_{\mathrm{mlf}}^{\mathrm{LW}}$, and enhanced overall repulsive forces with protein foulants. This energy profile originates from the hydrophilic modification layer rich in polar functional groups (e.g., –OH, –NH_2_) that form a robust hydration layer through strong hydrogen bonding with water molecules. This hydration barrier requires substantial energy for proteins to disrupt during membrane contact, effectively preventing foulant adhesion and enhancing fouling resistance. The xDLVO analysis provides quantitative thermodynamic evidence for the dramatically improved antifouling performance of the DA/TA/ZIF-8-UF membrane.

**Figure 6:**
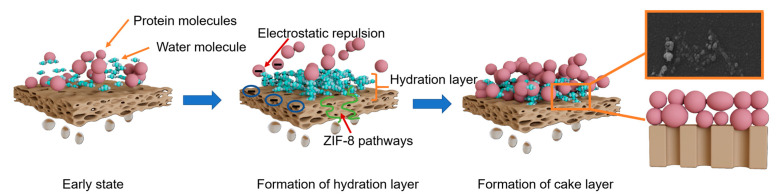
Mechanisms of antifouling properties and fouling model for DA/TA/ZIF-8-UF membrane.

Based on comprehensive membrane characterization and xDLVO analysis, the antifouling mechanism of the DA/TA/ZIF-8-UF membrane is illustrated in Figure 6. When protein-containing solutions contact the membrane surface, several synergistic effects occur:

(1) Hydration layer formation: The hydrophilic surface, rich in amino and phenolic hydroxyl groups, rapidly binds water molecules to form a dense hydration layer, preventing direct contact between proteins and the membrane surface and reducing initial adhesion.
(2) Pore size optimization: The metal-polyphenol networks (Zn^2+^-TA coordination) and DA/TA copolymerization create a tailored surface morphology that reduces effective pore size without significant permeability loss. This structure minimizes internal pore blockage by protein molecules while maintaining rejection performance.
(3) Enhanced water transport: The incorporated ZIF-8 crystals provide additional nanoporous pathways for water molecules, mitigating the permeability-selectivity trade-off and maintaining high flux during operation.
(4) Reversible fouling layer: As protein concentration increases during filtration, foulants primarily form a loose cake layer on the membrane surface rather than penetrating pores. This external fouling is more easily removed through hydraulic cleaning due to the hydration layer’s lubricating effect and electrostatic repulsion from the negatively charged surface.

The combination of these mechanisms, hydration barrier effect, optimized pore structure, enhanced water transport, and reversible fouling formation, explains the superior antifouling performance and stability of the DA/TA/ZIF-8-UF membrane compared to conventional PVDF membranes.

## 4. Conclusions

This study demonstrates a novel and efficient strategy for fabric high-performance antifouling PVDF hollow fiber ultrafiltration membranes through a metal-ion mediated co-deposition of dopamine (DA), tannic acid (TA), and ZIF-8 nanoparticles. The key findings can be summarized as follows:

First, the Zn^2+^-assisted coordination-driven self-assembly enabled the formation of a uniform, stable, and firmly adherent composite coating on the membrane surface under mild conditions. The metal-phenolic networks between Zn^2+^ and TA not only enhanced the interfacial adhesion but also facilitated the oriented growth of ZIF-8 crystals, creating a robust modification layer. Second, the modified membrane exhibited significantly enhanced surface hydrophilicity, as evidenced by the remarkably reduced water contact angle (21°) and increased surface negativity (zeta potential: −29.68 mV). The abundant polar functional groups (–OH, –NH_2_) facilitated the formation of a dense hydration layer, which was quantitatively verified by xDLVO analysis showing substantially increased interfacial free energy and stronger repulsive interactions with protein foulants. Third, the DA/TA/ZIF-8-UF membrane achieved exceptional separation performance with a high water permeability of 771 L/(m^2^∙h∙bar) and outstanding BSA rejection of 97.7%. The incorporated ZIF-8 crystals provided additional water transport channels while maintaining molecular selectivity, effectively mitigating the permeability-selectivity trade-off. Fourth, the membrane demonstrated superior antifouling capability against various protein foulants (BSA, LYS, OVA), with flux recovery rates exceeding 71.5% after fouling-cleaning cycles. The antifouling mechanism was attributed to the synergistic effects of hydration layer formation preventing direct foulant–membrane contact, optimized pore structure minimizing internal fouling, enhanced water transport through ZIF-8 pathways, and electrostatic repulsion from negatively charged surface groups.

This work provides valuable insights into the rational design of high-performance antifouling membranes through multifunctional surface engineering, offering promising potential for sustainable water treatment and bioprocessing applications.

## Figures and Tables

**Table 1:** Formula of the membrane modification solution.

Membrane	DA (g/L)	TA (g/L)	Zn^2+^ (mol/L)	H_mim_ (mmol/L)	Deposition Time (h)
DA-UF	1	-	-	-	6
TA-UF	-	4	-	-	6
DA/TA-UF	1	4	-	-	6
DA/ZIF-8-UF	1	-	0.015	0.7	6
TA/ZIF-8-UF	-	4	0.015	0.7	6
DA/TA/ZIF-8-UF	1	4	0.015	0.7	6

**Table 2:** Atomic percentages of surface elements on membrane surfaces from XPS analysis.

Membrane	C	N	O	F	Zn
PVDF substrate	58.23	0.97	6.87	33.94	-
DA/TA-UF	67.98	3.93	27.44	0.66	-
DA/TA/ZIF-8-UF	69.44	17.75	6.12	0.55	6.15

**Table 3:** Surface free energy of membrane and three pollutants.

**Materials**	$\gamma^{\mathrm{LW}}$	$\gamma^{+}$	$\gamma^{-}$	**γ^AB^**	**γ^TOT^**
Commodity UF	33.65	0.11	2.33	1.01	34.66
DA/TA/ZIF-8-UF	38.18	0.42	64.97	10.45	48.63
BSA	43.22	1.065	47.68	14.25	57.47
OVA	35.57	0.41	50.76	9.12	44.69
LYZ	26.71	0.93	28.31	10.26	36.97

**Table 4:** Interaction energies of membrane and protein pollutants.

	$\Delta G_{\mathrm{mlf}}^{\mathrm{LW}}$	$\Delta G_{\mathrm{mlf}}^{\mathrm{AB}}$	$\Delta G_{\mathrm{mlf}}^{\mathrm{EL}}$	$\Delta G_{\mathrm{mlf}}^{\mathrm{TOT}}$	$\Delta G_{\mathrm{SlS}}$
Commercial UF-BSA	−4.31	24.94	−0.00080	20.63	-
DA/TA/ZIF-8-UF-BSA	−5.75	40.51	−0.00080	34.76	-
BSA-BSA	−2.15	39.77	-	-	37.62
Commercial UF-OVA	−2.93	27.12	−0.00053	24.19	-
DA/TA/ZIF-8-UF-OVA	−3.91	44.82	−0.00053	40.90	-
OVA-OVA	−1.83	52.36	-	-	50.53
Commercial UF-LYZ	−1.13	−36.77	−0.00137	−37.90	-
DA/TA/ZIF-8-UF-LYZ	−1.51	−26.98	−0.00137	−28.49	-
LYZ-LYZ	−3.74	−6.52	-	-	−10.26

## Data Availability

The raw data supporting the conclusions of this article will be made available by the authors on request.

## Supplementary Materials

The following supporting information can be downloaded at: https://www.mdpi.com/article/10.3390/membranes15090277/s1, Figure S1: (a) Photo images of membranes and SEM cross-sectional images for the membranes of (b) PVDF substrate, (c) TA-UF, (d) DA-UF, (e) DA/TA-UF, (f) TA-ZIF-8-UF, (g) DA/ZIF-8-UF, and (h) DA/TA/ZIF-8-UF; Figure S2: High resolution SEM image of the ZIF-8 crystallites on the surface of (a) DA/TA-UF and (b) DA/TA/ZIF-8-UF membrane; Figure S3: ATR-FTIR spectra of membranes in the wavenumber of 2800-3600 cm^−1^; Figure S4: O1s peak fitting of (a) DA/TA-UF membrane and (b) DA/TA/ZIF-8-UF membrane; Figure S5: Correlation of permeability-porosity for membranes; Table S1: Properties of protein molecules and rejection rate by DA/TA/ZIF-8-UF membrane; Table S2: Contact angle and Zeta potential of membranes and different protein pollutants; Table S3 The surface tension parameters of the three testing agents.
