# Supplementary material for: Zn2+-Mediated Co-Deposition of Dopamine/Tannic Acid/ZIF-8 on PVDF Hollow Fiber Membranes for Enhanced Antifouling Performance and Protein Separation
Source: Membranes (Basel). 2025 Sep 15;15(9):277. doi: 10.3390/membranes15090277 (PMC12471975; doi:10.3390/membranes15090277)
Supplement: Supplementary file 1 [file membranes-15-00277-s001.zip › membranes-3859494-supplementary.pdf]

Supplementary information

Zn^{2+}-Mediated Co-deposition of Dopamine/Tannic Acid/ZIF-8 on PVDF Hollow Fiber Membranes for Enhanced Antifouling Performance and Protein Separation

Lei Ni[a], Qiancheng Cui[b], Zhe Wang[b], Xueting Zhang[b], Jun Ma[c], Wenjuan Zhang[b, *], Caihong Liu[d, *]

[a] *School of Material Science and Engineering, Tiangong University, Tianjin 300387, China*

[b] *Tianjin Key Laboratory of Aquatic Science and Technology, School of Environmental and Municipal Engineering, Tianjin Chengjian University, Tianjin 300384, China*

[c] *State Key Laboratory of Urban Water Resource and Environment, Harbin Institute of Technology, Harbin 150090, China*

[d] *Key Laboratory of Eco-environments in Three Gorges Reservoir Region, Ministry of Education, College of Environment and Ecology, Chongqing University, Chongqing 400044, China*

[*] Corresponding author. Tel.: +86 22 23085117; fax: +86 22 23085117.

E-mail addresses: wenjuanvivian@126.com (Wenjuan Zhang), caihong.liu@cqu.edu.cn (Caihong Liu)

Figure S1

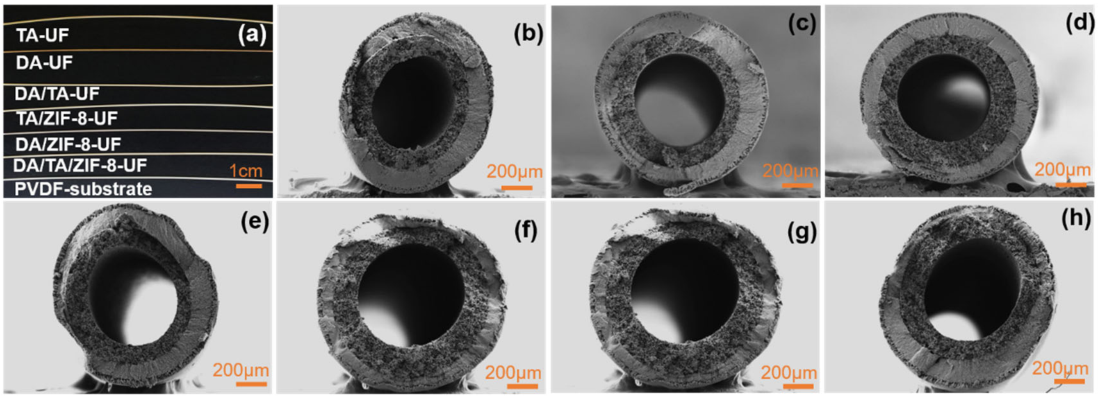

Figure S1 (a) Photo images of membranes and SEM cross-sectional images for the membranes of (b) PVDF substrate, (c) TA-UF, (d) DA-UF, (e) DA/TA-UF, (f) TA-ZIF-8-UF, (g) DA/ZIF-8-UF, and (h) DA/TA/ZIF-8-UF.

Figure S2

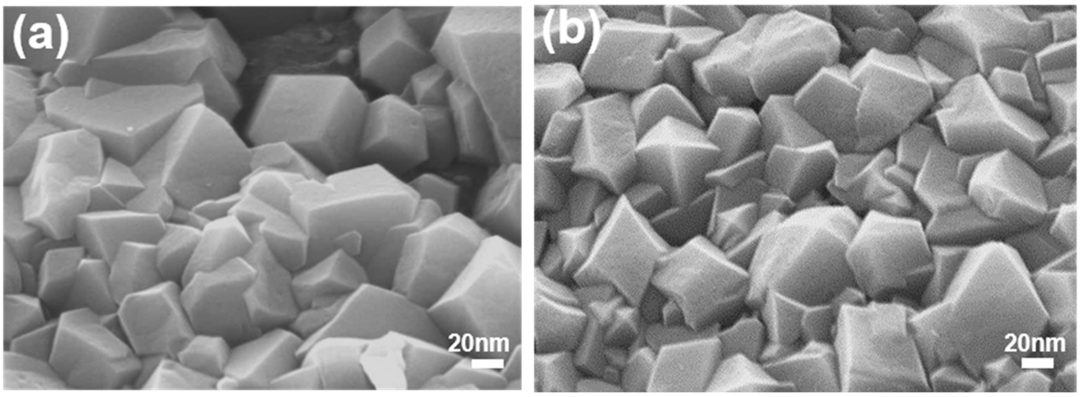

Figure S2 High resolution SEM image of the ZIF-8 crystallites on the surface of (a) DA/TA-UF and (b) DA/TA/ZIF-8-UF membrane.

Figure S3

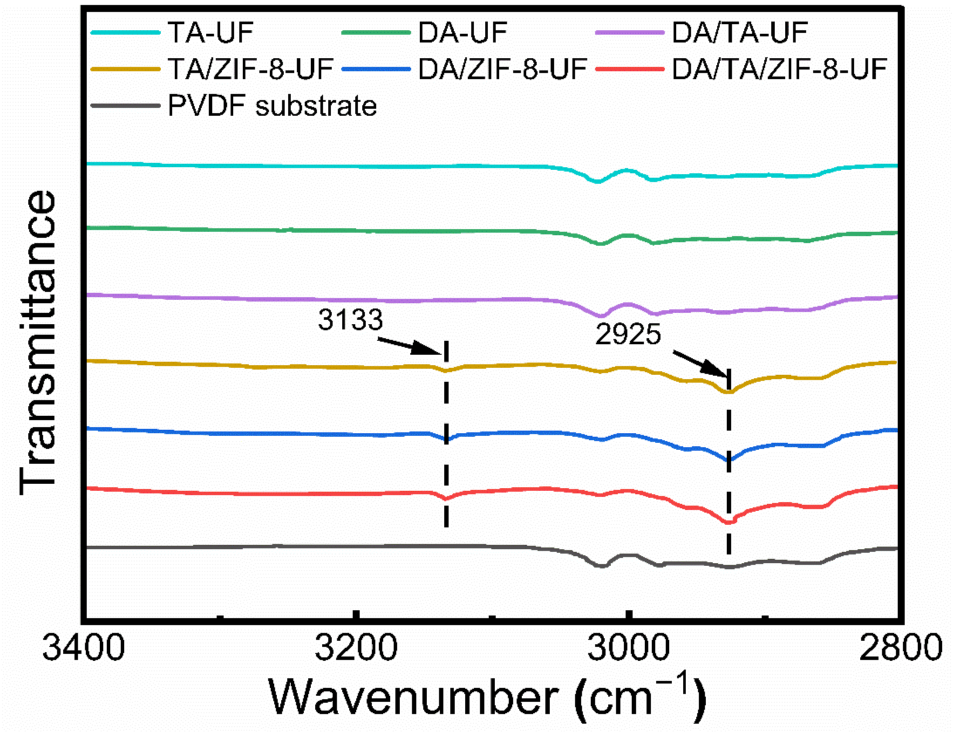

Figure S3 ATR-FTIR spectra of membranes in the wavenumber of 2800-3600 cm^{-1}.

Figure S4

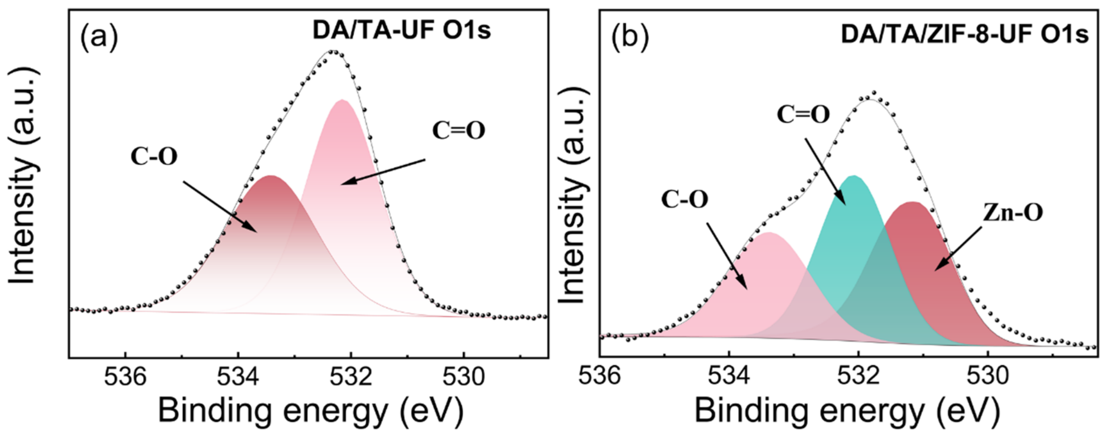

Figure S4 O1s peak fitting of (a) DA/TA-UF membrane and (b) DA/TA/ZIF-8-UF membrane.

Figure S5

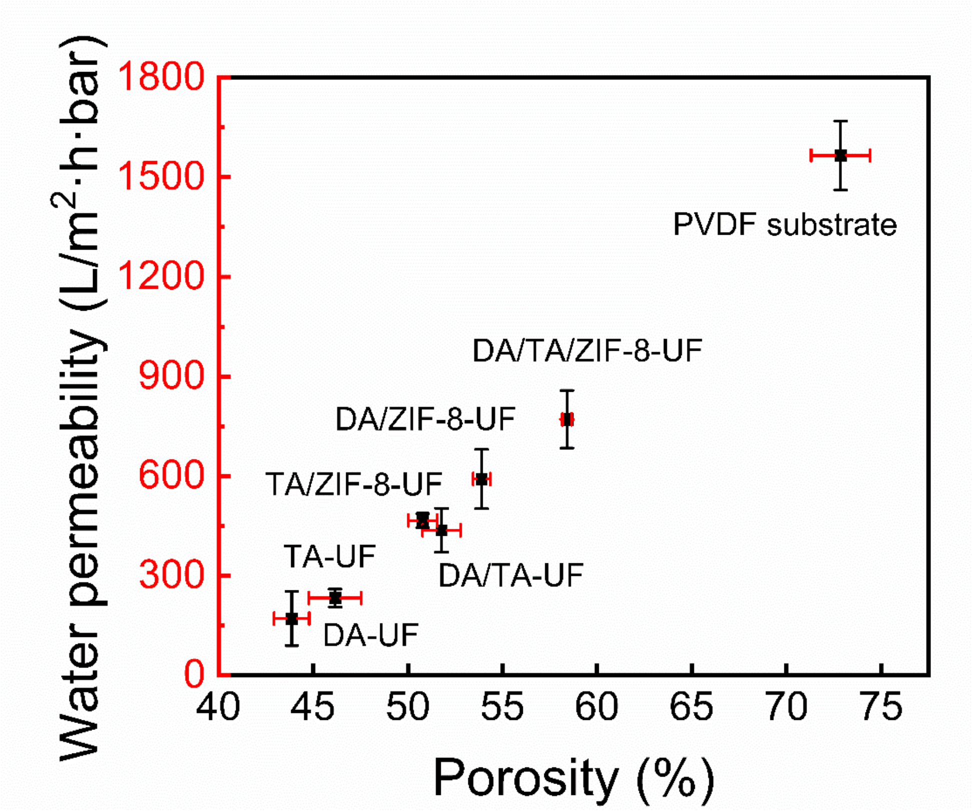

Figure S5 Correlation of permeability-porosity for membranes.

Table S1 Properties of protein molecules and rejection rate by DA/TA/ZIF-8-UF membrane

Proteins	Molecular formula	Molecular weight	Absorption wavelength(nm)	Electrically charged	Rejection rate (%)
BSA	$(C_8H_{21}NOSi_2)_n$	68 kDa	280	negative	97.7±0.2
OVA	$(C_{16}H_{26}O_5)_n$	44.5 kDa	230	negative	82.5±0.3
LYS	$(C_{11}H_{20}NO_6)_n$	14-16 kDa	281	positive	71.4±0.5

Table S2 Contact angle and Zeta potential of membranes and different protein pollutants.

Materials	θ_W (°)	θ_G (°)	θ_D (°)	Zeta potential (mV)
Commercial UF	95.23 (±0.8)	66.21 (±2.4)	50.73 (±1.2)	-19.83 (±2.7)
DA/TA/ZIF-8-UF	20.61 (±0.4)	53.35 (±1.6)	42.33 (±0.3)	-29.68 (±3.3)
BSA	50.00 (±1.3)（	42.55 (±2.0)	31.72 (±0.8)	-38.8 (±2.6)
OVA	97.22 (±4.6)	81.32 (±3.4)	47.25 (±2.8)	-4.40 (±0.3)
LYZ	79.24 (±0.8)	69.47 (±1.2)	62.94 (±1.3)	+4.95 (±0.7)

Table S3 The surface tension parameters of the three testing agents.

Test reagent	γ^{LW}	γ^{+}	γ^{-}	γ^{TOT}
Pure water	21.8	25.5	25.5	72.8
Glycerol	34.0	3.9	57.4	64.0
Diiodomethane	50.8	0.0	0.0	50.8
